# Research progress of the application of mesenchymal stem cells in chronic inflammatory systemic diseases

**DOI:** 10.1186/s13287-021-02613-1

**Published:** 2022-01-08

**Authors:** Fangfang Huang, Erick Thokerunga, Fajian He, Xinyu Zhu, Zi Wang, Jiancheng Tu

**Affiliations:** 1 Program and Department of Clinical Laboratory Medicine, Center for Gene Diagnosis, Zhongnan Hospital of Wuhan University, Wuhan, 430071 China; 2 Department of Radiation and Medical Oncology, Zhongnan Hospital of Wuhan University, Wuhan, 430071 Hubei China

**Keywords:** Mesenchymal stem cells, Prognosis, Biomarkers, Rheumatoid arthritis, Systemic lupus erythematosus, Inflammatory bowel disease

## Abstract

Chronic inflammatory systemic diseases are the result of the body's immune imbalance, with a long course and recurring episodes. Immunosuppressants are the main treatment, but not all patients respond well to it. Being capable of both self-renewal and differentiation into multiple tissue cells and low immunogenicity, mesenchymal stem cell is a promising treatment for chronic inflammatory systemic diseases. In this article, we describe the research progress and clinical application of mesenchymal stem cells in chronic inflammatory systemic diseases and look for influencing factors and biomarkers that can predict the outcome of patient with mesenchymal stem cell transplantation.

## Background

Chronic inflammatory systemic disease (CID) is a collective term for diseases that are characterized by prolonged inflammation lasting several months to years. They include rheumatoid arthritis, inflammatory bowel disease, systemic lupus erythematosus, etc. Due to a damaged inflammation self-regulatory mechanism, patients with CID have repeated episodes of inflammation, resulting into simultaneous destruction and healing of tissues at the inflammation site [1]. Corticosteroids, immunosuppressants and monoclonal antibodies are used to treat immune diseases, but drug resistance and adverse reactions limit their use. As stem cell therapy becomes more have gained attention due to their ease to obtain and lesser ethical requirements.

The International Society for Cell Therapy defines mesenchymal stem cells (MSC) as non-hematopoietic stem cells that are: (1) plastic adherent in standard culture conditions; (2) express CD73, CD90 and CD105 but lack CD11b, CD14, CD34, CD45 CD19 or CD79α and HLA-DR antigens, and (3) differentiate into osteoblasts, adipocytes and chondroblast in vitro [2]. Mesenchymal stem cells are found in the bone marrow, umbilical cord blood, the placenta, adipose tissues, amniotic fluids, dental tissues, skin, hair follicles and tonsils [3].

**Fig. 1:**
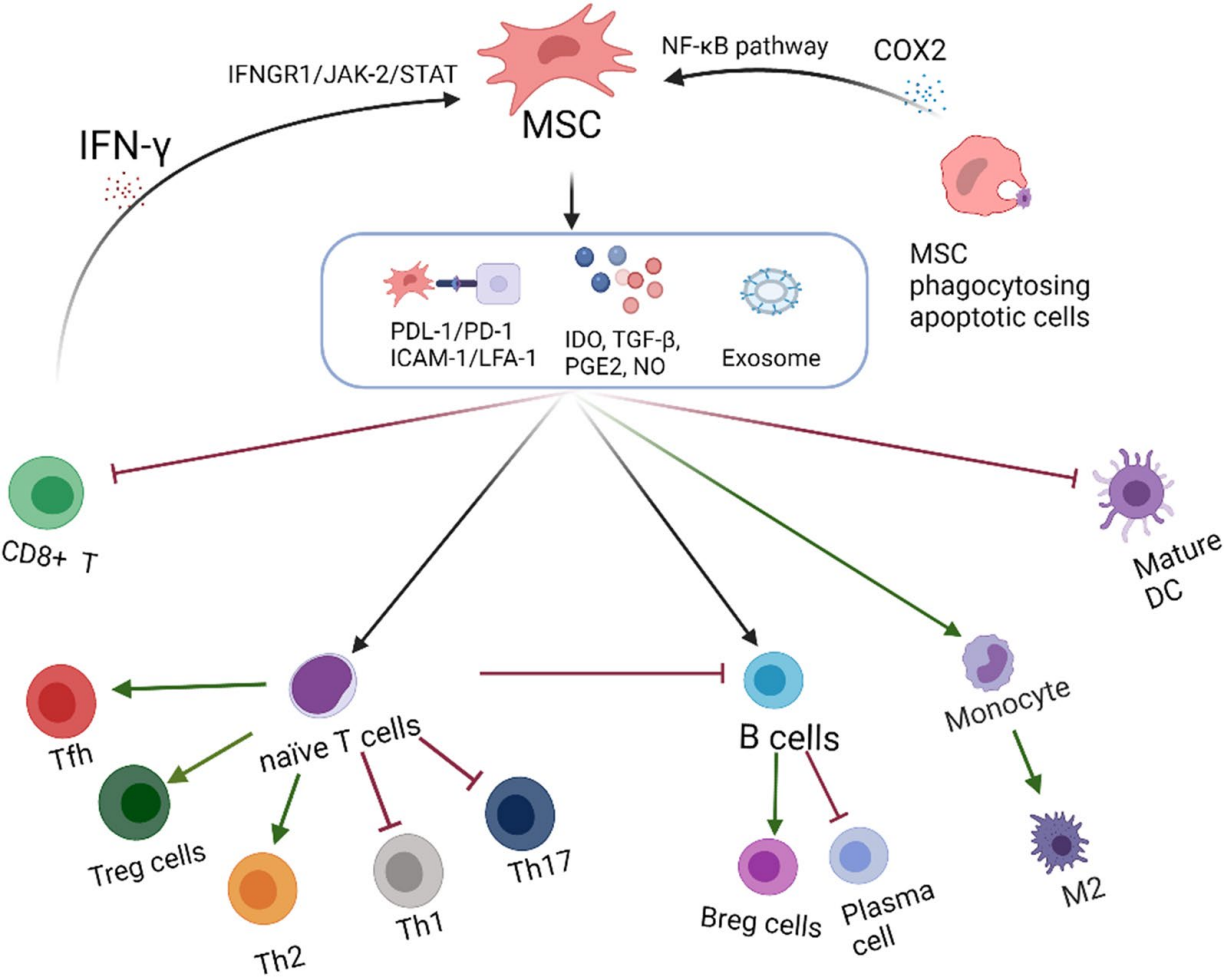
The immunosuppressive mechanisms of MSC MSCs activated by IFN-γ and other inflammatory factors regulate a variety of immune cells through secreting IDO, TGF-b, NO, PGE2 and other molecules, exosomes, and cell–cell connection, including T, B, NK and Macrophages. Among which T cells are the main target cells of MSC immunosuppression, can inhibit the polarization of naive T cells to pro-inflammatory cells Th1 or Th17, Tfh, and promote the differentiation of naive T cells to regulatory immune cells Treg, and indirectly through T cells Inhibit the proliferation and differentiation of B cells. In addition, MSC can directly inhibit the differentiation of B cells into plasma cells, promote the differentiation of B cells into Breg cells. MSCs promote the polarization of macrophages to the inflammation-suppressing phenotype M2, and inhibit maturation of DC

Mesenchymal stem cells are pluripotent progenitor cells capable of migration to injury and tumor sites, self-renewal and differentiation into multiple tissues. They are strong immune modulators producing immunomodulatory molecules such as indoleamine 2,3-dioxygenase (IDO) [4], TGF-β [5], PGE2 [6]and NO [7], or acting directly through PDL-1/PD-1 [8] PDL-1/B7-H1 [9] or ICAM-1/LFA-1 [10] ligand to influence immune cells’ proliferation, differentiation, maturation and polarization [11, 12]. The immunosuppressive mechanisms of MSC are shown in Fig. 1.

IFN-γ, a type II interferon, enhances both the immunosuppressive and migratory ability of MSC [13]. The IFN-γ produced by T cells promotes the expression of IDO in MSC through IFNGR1/STAT and p38-MAPK signaling [4, 14, 15]. Two types of IDO exist, IDO1 and IDO2. Autoreactive B cell responses are mediated by IDO2, while autoreactive T cell responses are indirectly affected by IDO1 expression [16]. IDO depletes tryptophan in local tissue microenvironments and generates kynurenines a immunoregulatory catabolites, thus activate GCN2 signaling pathway and mTOR signaling pathway [17, 18] and down-regulates the expression and activation of Vav1 protein [19]. This induces the conversion of naïve CD4(+)CD25(−) T cells into highly suppressive Treg; therefore, the modulation of IDO activity favors the interconversion between Treg cells and Th17 inflammatory cells [20]. Certain studies, however, show that IDO inhibitors do not have effects on Treg and Th17 cells [21]. IDO also induces increased PDL-1 expression and upregulates different immunomodulatory exosome-derived miRNAs that are involved in the control of both T cell activation/anergy and monocyte differentiation, resulting into inhibition of T cell activity, induction of M2-like phenotypes in monocytes, and increased production of IL-10 [4, 8].

TNF-α could enhance the expression of IFN-γ receptor (IFN-γR) via NF-κΒ signaling, in turn, enhanced responsiveness of MSC to IFN-γ stimulation activated STAT5 and p38-MAPK signaling [14]. However, studies have shown that TNF-α sometimes inhibits the immunosuppressive ability of MSC by enhancing the expression of costimulatory molecules ICOSL and HLA-DR on the surface of dendritic cells, while reducing the expression of PDL-1/PD-1, IL-10 and TGF-β [22]. Transforming growth factor beta (TGF-β) is a crucial cytokine for immune response regulation [23]. MSC secreted TGF-β skews macrophage polarization toward the M2-like phenotype through modulating the Akt/FoxO1 pathway, thus improving their phagocytic ability. It is also believed to up-regulate Treg cells [5, 21]. Phagocytosed apoptotic cells in turn release COX2 that promote MSC’s secretion of PGE2 resulting into down regulation of Th17 cells [6, 21]. Nitric oxide (NO) from MSCs is involved in the suppression of STAT5 phosphorylation and T follicular helper (Tfh) cell expansion [7, 24].

Recently, B cells have become a major target for the treatment of immune disorders. Results of MSC and B cell co-cultivation show that MSCs support static B cell survival but do not induce B cell proliferation. In addition, they regulate B-cell function through directly promoting B cells differentiation into IL-10 producing CD19+CD24hiCD38hi B cells, known as Brag cells; or by suppressing B cells via T helper or Tfh cells through repressing the differentiation of naive CD4(+) T cells into Tfh cells; or through releasing IDO and iNOS [7, 25–27].

In addition, studies have found that exosomes derived from mesenchymal stem cells also play an important role in mesenchymal stem cell therapy. A study of intervertebral disc degeneration found out that BM-MSC-derived exosomes promote the growth and survival of host cells. At the same time, nucleus pulposus cells-derived exosomes promoted BM-MSC migration and induced its differentiation to a nucleus pulposus-like phenotype [28]. MSC-derived exosomes also promote macrophages switch to M2 phenotype [29]. Interestingly, MSC can mischievously transfer its own mitochondria into Th17 cells and decrease their activity by increasing their oxygen consumption [30]. In diseases such as rheumatoid arthritis (RA) and systemic lupus erythematosus (SLE), where the phenotype and function of MSC of the patients differ from those of healthy ones, MSC can be targets for treatment [31–34].

MSCs are currently the most widely used stem cells in clinical practice despite the inconsistent clinical outcomes due to the difference in sources, dosages and timing [35]. This review seeks to answer the questions of which type of patients are suitable for MSC therapy, and what indicators can be used to monitor effectiveness of the treatment. Bone marrow and adipose tissues derived MSCs are popularly used, only a handful of studies have reported the use of placenta derived MSC. Studies also show that MSC pretreatment with IFN-γ or combined with IL-4/IL-25 has a better therapeutic effect [36–38]. In vitro studies have shown that highly inflammatory synovial fluid (SF) could better stimulate the proliferation and immunosuppressive ability of MSC [39], suggesting that MSC can achieve the best effect when the inflammatory factors in the patient's body are increased or right in the course of the disease. MSC delivery methods include systemic delivery and local delivery. Intra-venous delivery is the most commonly used route of administration [40]. However, MSCs injected intravenously are sometimes enriched in the lung and spleen tissues, where they are easily recognized and eliminated by immune cells, leaving only a small number to reach the targeted organs [41]. Most of the current clinical research patients receive a single intravenous infusion of 1 × 10^6^ cells/kg, and only a small part of patients will undergo two to three infusions. The local injection dose of colon tissue is 1 × 10^7^–1.2 × 10^8^ cells. Furthermore, a few dose-escalating trials have found no dose–response relationship [42, 43]. As shown in Table 1, intravenous infusion of MSC is relatively safe, and serious adverse reactions related to treatment are rare. However, some adverse events occasionally occur. Common adverse events in clinical studies include leukopenia, pneumonia, subcutaneous abscess, infection and fever. In refractory Crohn's disease, the incidence of adverse reactions of local MSC transplantation is relatively high, including anal abscess, fever, fever, fistula or anal bleeding, colon infection and so on.

**Table 1:** Trials about MSCs in RA, SLE, IBD

MSC source	No. of patients	Dosage and usage	Result	Adverse events	Indicator^a^	References
hUC- MSCs	63 RA	1 × 10^6^ cells/kg intravenous infusion	53.3% (MSCT), 93.3% (MSCT+IFN-γ) in 3-month follow-up	No safety issues in 1-year follow-up	**HAQ, DAS28, ESR, CRP, RF, Tregs/Th17** *anti- CCP*	[37]
UCMSCs	105 RA	1 × 10^6^ cells/kg intravenous infusion	53.85% in 3-month follow-up, last for 48 weeks	No serious adverse events	**HAQ, DAS28, platelet, Treg/Th17, IL-6, TNF-α** *anti-CCP, RF, IL-1β, IL-2R, IL-8* ***hemoglobin, albumin, IL-10, IFN-γ***	[58]
adMSCs	110 CIA animal model	1 × 10^6^/mouse intravenous infusion (tail vein)	14 days		**GM-CSF+CD4+T, Lag3+Tr1** ***Tregs, Tr1, (Tregs+Tr1)/GM-CSF+CD4+T***	[64]
hUCB-MSCs	9 RA	2.5 × 10^7^, 5 × 10^7^, 1 × 10^8^ cells intravenous infusion	4 weeks	no short-term safety concerns	**DAS28, IL-1β, IL-6, IL-8, TNF-α (in 1 × 10**^**8**^** group), ESR** *VAS, HAQ, ALT, ANC, AST,BUN,hs-CRP,MTX,WBC, Creatinine, Glucose, Triglyceride, Albumin, Albumin, Total protein* ***IL-10 (in 5 ×*** ***10***^***7***^*** group), Uric Acid***	[60]
UC-MSC	64 RA	4 × 10^7^ cells/RA intravenous injection	1 year 3 years	4% showed flu-like symptoms	**DAS28, HAQ, ESR, CRP, RF, globulin, Platelet (1 year, 3 year)** **anti-CCP (3 year)** *TP, ALB, WB, MCV*	[61]
BMSC	30 RA	4 × 10^7^cells /joint intra-articular knee implantation	12 months	pain /articular swelling and other minor adverse events	**WOMAC, time to jelling and pain-free walking distance, MTX and prednisolone intake** *DAS 28, ESR, CRP, MRI imaging score*	[62]
BM-MSCs/ UC-MSC	81 SLE	1 × 10^6^ cells/kg intravenous infusion	34% (remission for 5 years) 84% (survival rate for 5 years) 24% (relapse within 5 years)	renal dysfunction, diarrhea, infection, myocardial infarction, diabetes	**proteinuria** *C4, serum urea nitrogen, creatinine, and uric acid* ***albumin, C3, WBC, hemoglobin, platelet***	[59]
BM-/UC-MSCs	69 SLE	1 × 10^6^ cells/kg intravenous infusion	58%(LDA) 23%(remission)			[80]
hUC-MSC	18 LN	2 × 10^8^ cells intravenous infusion	75%(remission)	leucopenia, pneumonia, subcutaneous abscess		[82]
BM-/UC-MSCs	35 SLE	1 × 10^6^ cells/kg intravenous infusion	24 months	no adverse events	**SLEDAI, Th17** ***WBC, platelet,*** ***Hb, Tregs, Treg/Th17***	[79]
hBM-MSCs	NZB/W mice	1 × 10^6^ cells/mouse/injection at 17, 19, and 21 weeks of age etro-orbital injection of the venous sinus			**anti-dsDNA** *urinary albumin, Th 1Th2, Th17, Tregs*	[27]
UC-MSCs	30SLE	1 × 10^6^ cells/kg intravenous infusion	12 months		**Th17,IL-17,TNF-α,** *IL-6, IL-17A* ***Treg, Foxp3, TGF-β***	[21]
adMSC/iMSC	DSS	1 × 10^6^ cells at 10, 13, 16 day tail vein infusion			**F4/80+, CD11b+macrophages** **CD103+monocytes** *CD3*+*T cells, CD4*+*T cells* ***Ki-67*+*intestinal epithelial cells*** ***LGR5*+*intestinal stem cells*** ***CD31*+*endothelium FOXP3*+*Tregs*** (*in tissue*)	[41]
hBM-MSCs	IL-10 − / − mice	5 × 10^5^ cells 0, 1 week tail vein infusion			**ROS, lipid MDA formation, INF-γ, TNF-α, IL-4, CD8, p-NF-kB, type I collagen,** *CD4, SOD2* ***Catalase, SOD1*** (*in tissue*)	[99]
hBM-MSCs	DSS	5 × 10^6^ cells 1, 2, 3 day tail vein infusion			**IL17A**^**+**^** Th17, IFN-γ**^**+**^** Th1** ***FOXP3*+*Tregs, IL4*+*Th2*** (*in PB, tissue*) ***Ki-67+intestinal epithelial cells*** ***LGR5+intestinal stem cells*** ***CD31+endothelium*** (*in tissue*)	[38]
IBM-MSCs	DSS	1 × 10^6^ cells 7 day injected intraperitoneally			**IL-6, TNF-α, IFN-γ, IL-17A** *IL-10* (*in tissue*)	[13]
IBM-MSCs	TNBS	1 × 10^6^ cells 7 day injected intraperitoneally			**SAA, TNF-α, IL-6,** *IFN-γ* ***IL-17A, IL-10*** (*in tissue*)	[13]
adMSCs	TNBS	1 × 10^6^ cells 1, 2 day injected intraperitoneally			**TNF-α, IFN-γ, IL-6, IL-1β, and IL-12, RANTES, macrophage inhibitory protein 2,Th1** ***IL-10, Treg*** (*in tissue*)	[100]
adMSCs	TNBS	3 to 5 × 10^6^ cells injected intraperitoneally			**TNF-α, IL-12, IL-6, IL-23, IL-21, IFN-γ** *IL-17A* ***IL-10, TGF-β*** (*in serum*) **Th1, Th17** *Th2* ***CD5*+*Breg*** (**in spleen, MLN**)	[101]
P-MSCs	EF	1 × 10^6^ cells intralesional injection			**IL-1β, IL-6, TNF-α, IFN-γ, ROS** ***IL-10, TGF-β, VEGF, Ang-2*** (*in tissue*)	[98]
adMSCs	24 CD with fistulas	2 × 10^7^ cells intralesional injection	At 24 weeks 69.2% (response) 56.3% (some fistulas close) 30% (all fistula close)	anal abscess (12.5%) pyrexia (4.17%) uterine leiomyoma (4.17%)	**MSS, PDAI** *CDAI*	[103]
BM-MSCs	15 CD with fistulas	1 × 10^7^, 3 × 10^7^, 9 × 10^7^ cells intralesional injection	At 12 weeks 40.0%, 80.0%, 20.0% (all fistula close) At 24 weeks 80.0% (1 × 10^7^) Follow-up by 4 years 63.0%, 100%, 43.0% (closed fistula)	Fever anal pain pus blood from the fistula or anus	**PDAI, IL-8, IL-1β, IL-6** *IL- 10, TNF, IL-12p70 (cannot be detected)*	[42, 102]
autologous MSC	12 CD with fistulas	2 × 10^7^ cells intralesional injection	At 24 weeks 83% (all fistula close)	no related adverse events		[105]
autologous ADSVF	10 CD with fistulas	intralesional injection	At 12 weeks 20% (combined remission), 70% (clinical response) At 48 weeks 60% (combined remission), 80% (clinical response)	Flares fistula tract	**PDAI** *CRP, fibrinogen, WBC* *SIBDQ*	[106]
adMSCs	212CD with fistulas	1.2 × 10^8^ cells intralesional injection	50% (remission) 17% (adverse events)	anal abscess proctalgia	**PDAI** *IBDQ, CDAI, time to combined remission, relapse, time to relapse, van Assche score*	[104]
BM-MSCs	12CD	2 × 10^6^, 5 × 10^6^, 10 × 10^6^ cells/kg intravenous infusion	12-weeks	acute appendicitis, C. difficile colitis		[43]
UC-MSCs	82 CDAI 220–450	1 × 10^6^ cells/kg intravenous infusion	No patient achieved complete remission (CDAI < 150)	upper respiratory tract infection	**CDAI, HBI, and corticosteroid dosage** *Blood cell count. Liver and renal function*	[107]
BM-MSCs	13 CDAI 220–450	1.5 to 2.0 × 10^6^ cells/kg at weeks 0 and 4 intravenous infusion	At week 12 15.4% (clinical response) 7.7%(remission)	no related adverse events	*CDAI, CRP, FC, Treg, CD4*+*T, CD8*+*T, B, IgA/G/M* ***NK%, NKT%***	[96]

Bold indicates a decrease, italic indicates no significant change, and bold italic indicates an increase

Given that the traditional managements of CID (anti-inflammatory drugs/hormone therapy) do not benefit all patients, it is no surprise that MSC is being explored as a possible therapeutic alternative. However, we still know very little known about what happens when MSC are injected into the patient. This has made it difficult to associate with certainty their action to the healing of chronic inflammatory processes. Identification of biomarkers that are associated with the action of MSC and their healing property on CID is extremely vital. This will enable objective assessment of the effectiveness of MSC therapy in chronic inflammatory systemic diseases and monitoring of any side effects thereof. In this mini review, we discuss the progress made in the mesenchymal stem cell therapy of Rheumatoid Arthritis (RA), Systemic Lupus Erythematosus (SLE), Inflammatory Bowel Disease (IBD) and explore the clinically significant biomarkers that are associated with their prognosis.

## Rheumatoid arthritis (RA)

Rheumatoid arthritis (RA) is a chronic inflammatory autoimmune disease characterized by synovial hyperplasia and edema. Its sequelae involves inflammatory cell infiltration of the synovium, cartilage damage and bone erosion due to the chronic inflammatory process [44]. A major factor in RA pathogenesis is the inflammation of intra-joint connective tissue called synovium. The inflammatory process is composed mostly of fibroblast-like synoviocytes (FLS), macrophages and infiltrating lymphocytes [45], with the macrophages maintained in a delicate back and forth transition between pro-inflammatory M1 and anti-inflammatory M2 phenotypes [46]. Bone destruction is closely related to the imbalance between osteoclasts and osteoblasts. Intracellular signaling pathways such as MAPK, Wnt, Hedgehog (Hh), Notch, Akt/mTOR, TGF-β/BMP are involved in regulating the proliferation and differentiation of osteoblasts. Highly inflammatory synovial fluid inhibits osteoblast proliferation [47]. Osteoclastogenesis needs two factors, macrophage colony-stimulating factor (M-CSF) for proliferation and survival, and receptor activator of NF-κB ligand (RANKL) for differentiation and function [48]. RANKL is a TNF superfamily member and an essential mediator of osteoclastogenesis, produced by osteoblasts, synovial fibroblasts and activated T cells [48]. Only Th17 has been confirmed to be associated with proliferation and differentiation of osteoclasts in RA, thus promoting bone destruction [49].

Patient Activity Scale (PAS) or PASII, Routine Assessment of Patient Index Data 3 (RAPID-3), Clinical Disease Activity Index (CDAI), Disease Activity Score with 28-joint counts (ESR or CRP), Simplified Disease Activity Index (SDAI) are instruments to measure rheumatoid arthritis disease activity and to define remission [50]. RF (rheumatoid factor), ACPAs (anticitrullinated protein antibodies), ESR (erythrocyte sedimentation rate) and CRP (C-reactive protein) are used as RA classification criteria in clinical practice [51]. RA patients also present increase in other autoantibodies such as anti-carbamylated proteins, anti-peptidylarginine deiminase (PAD-4), anti-collagen type II, and anti-IgG hinge, as well as a variety of inflammatory factors, chemokines [52].

For RA disease, in addition to the immunosuppressive function of MSC, other therapeutic mechanisms of MSC have also been explored. Recent pre-clinical study has discovered that hUCB-MSCs can promote the transition of macrophages tilting the equilibrium toward the formation of M2-type cells by the tumor necrosis factor (TNF)-α-mediated activation of cyclooxygenase-2 and TNF-stimulated gene/protein 6 in hUCB-MSCs, a process that favors cartilage production and thus repair of inflammatory damages in RA [53]. In addition, MSC were observed in the cartilage tissue from day 11 and until 42 days after intravenous injection and differentiated into cartilage and osteoblasts via MAPK and Wnt signals [54–56], while partially inhibiting osteoclast formation via CD39-CD73-adenosine signals [57], thereby preventing bone destruction.

As a therapeutic agent, the combination of MSC with IFN-γ has been shown to significantly alleviate symptoms of arthritis in up to 93.3% of patients within 3 months, and no relapse within 1 year of follow-up [37]. It has been discovered that high concentrations of IFN-γ produced by T cells induce MSC to produce more indoleamine 2,3-dioxygenase (IDO), thereby enhancing MSC's autoimmune regulation ability [15]. IFN-γ thus maybe a key moderator of MSC’s therapeutic function in RA patients whose serum level is directly associated with the RA patients’ response to MSC therapy. A study by Yang et al. [58] assessed possible serum biomarker for predicting the therapeutic effect of MSC therapy in rheumatoid arthritis (RA) patients and found out that MSCT resulted in a transient increase in serum IFN-γ (> 2 pg/ml), promoted an increase in IL-10 levels and the Treg/Th17 ratio, and decrease DAS28, the decreased value at the 12-week was closely related to the increase in IFN-γ level. In a work, a high level of serum IFN-γ before or transient rise in IFN-γ after mesenchymal stem cell transplantation is a positive predictor of RA remission.

A 5-year follow-up found that patients had 34% of remission, 84% of survival rate, and 24% of recurrence rate [59]. Intravenous MSC transplantation (MSCT) significantly decreased DAS28, HAQ, platelets, ESR, CRP, RF, prednisone dose, antinuclear antibody (ANA), cartilage oligomeric matrix protein (Comp), tissue inhibitor metalloproteinase-1 (Timp1), matrix metalloproteinase 1 (Mmp-1) and IL-1R, MCP-1, IL-6, TNF-α, increase hemoglobin, albumin, Treg/Th17 ratio, IL-10, IFN-γ, and the proportion of Low Disease Activity [36, 58, 60], while the result of IL-1β, IL-8, IL-2R, anti-CCP antibody, RF is inconsistent [60, 61]. Liver, kidney function and immunoglobulins levels from each patient were all within normal range before, 1 year and 3 years after UC-MSC treatment; HAQ and DAS28 continued to decrease at 3 years after treatment, suggesting the long-term efficacy of UC-MSC treatment [61]. 6.7% patients in the MSCT group relapsed at the 24th week of follow-up accompanied by elevated levels of ESR and CRP [37]. However, improvement of WOMAC, VAS, time to jelling and pain-free walking distance could not be significantly sustained beyond 12 months, and the MRI imaging score of the knee did not reveal any improvement in some of the patients who received BMSCs by intra-articular knee implantation [62].

Recent data have identified that GM-CSF-expressing T cell is a unique T helper subset having critical roles in the pathogenesis of arthritis and other inflammatory diseases [63]. Lopez-Santalla et al. [64] conducted an experiment in arthritis mice to examine the modulation effect of MSC on GM-CSF CD4+T cells and Th17 cells. They observed a significant decrease in the severity of the arthritis shortly after injecting the mice with adipose-derived MSC. A further notable effect was the reduction in the number of pathogenic GM-CSF CD4+T cells in the spleen and peripheral blood, accompanied by an increase in the number of Treg and IL10+IL17−CD4+T cells in the draining lymph nodes [64]. It is clear that MSCT induces changes in different types of immune cells in peripheral blood and so the best option is to monitor a variety of immune cells at ago in order to accurately determine treatment outcomes following MSCT. Changes in the number of immune cells in the spleen, however, seem more accurate and should be explored further.

In RA, therefore, MSC combined with IFN-γ significantly alleviate symptoms of the disease with good long-term outlook (No relapse up to 1-year post treatment). Moreover, a high level of serum IFN-γ before or transient rise in IFN-γ after mesenchymal stem cell transplantation positively predicts good treatment outcome. MSC can reverse some of the clinical evaluation indicators of RA patients, such as DAS28, HAQ and immune markers, and reduce joint tissue damage markers, but the changes in autoantibodies after MSCT are not obvious. ESR and CRP are good biomarkers for monitoring MSCT therapy. Since immune markers are non-specific, monitoring a variety of them at once is a better indicator of prognosis and treatment outcome.

## Systemic lupus erythematosus (SLE)

SLE is an autoimmune disease characterized by the destruction of the patient’s autoimmune tolerance, production of nuclear-antibodies and immune complexes, and disruption of multiple organ functions. 10% of SLE patients eventually develop lupus nephritis (LN), and only half of the LN patients recover [65]. The target for managing lupus is to improve patients’ long-term outcomes and quality of life, and so management plan is to treat the disease symptoms, prevent damage to other major organs and minimize drug side effects. The pathogenesis of SLE is multifactorial. Abnormal clearance of apoptotic cells is related to the beginning of SLE [66]. Autoantigens are released mainly from secondary necrotic cells because of a defective clearance of apoptotic cells or an inefficient degradation of DNA, and then, these autoantigens are presented by dendritic cells to autoreactive B cells, forming immune complexes (IC). Many patients with systemic autoimmune diseases including SLE have signs of aberrant production of type I interferon (IFN) and display an increased expression of IFN-inducible genes, and the clearance of antinuclear IC via Fc-gamma receptors is considered a central event in amplifying inflammatory immune responses in SLE [66, 67]. Excessive activation of B cells and the production of autoantibodies play an important role in SLE. Current studies have suggested that both impairments of Breg cell functions and expansions of autoreactive B subsets (Age-Associated B cell, inate-like B cells, plasma cells) lead to immune tolerance breakdown and autoimmune progression [68].

Diagnostic biomarkers frequently used in clinical practice include ANA, anti-dsDNA antibodies, anti-Sm, anti-cardiolipin, anti-β2-glycoprotein I, lupus anticoagulant, complement proteins C3 and C4(↓) [69]. SLE Disease Activity Index (SLEDAI), the dosage of immunosuppressive agents, anti-dsDNA antibodies, serum complement proteins C3 and C4 are usually applied to SLE assessment while creatinine clearance (↓), urine protein (↑), and lung volume (↓) are applied to organ function monitoring [70]. Potential biomarkers for lupus disease activity include anti-C1q antibodies, CBCAPs (RC4d, EC4d and EC3d), IFN-a and IFN-inducible genes, B-cell-activating factor (BAFF) or B-lymphocyte stimulator (BlyS), a proliferation inducing ligand (APRIL) [71]. CD4+ and CD8+T cell transcription signals, Treg, Tph (T peripheral helper) cells, IFN, cfDNA, CTHRC1 (collagen triple helix repeat containing 1) are closely related to SLE outcome and SLEDAI [72–77].

Mesenchymal stem cell therapy (MSCT) is safe and results into long-term clinical remission in SLE patients. A five-year follow-up study found that 34% of patients with SLE had remission, 84% survival rate, and recurrence rate 24% post MSCT [59]. SLE Disease Activity Index (SLEDAI) scores decreased significantly, while albumin, complement C3, WBC, platelets, hemoglobin, lung volume and quality of life continued to improve during follow-up; dsDNA, ANA, proteinuria, serum urea nitrogen, creatinine levels decreased in 1 year after MSCT [59, 78]. The number of Treg, Treg/Th17 ratio, Foxp3 and TGF-β in the MSCT group increased, while Th17, IL-17 and TNF-α decreased significantly, and IL-6 and IL-17A had no changes [21, 79]. Pretreatment factors that can affect treatment efficacy are discussed by Wen et al. [80]. In a separate study, there was no difference in most of the above immune markers between the treatment and control group. SLEDAI score, blood cell count, serum albumin, proteinuria and Treg/Th17 percentages between those with or without cyclophosphamide (CYC) pretreatment, those infused with bone marrow- or umbilical cord-derived MSCs were similar between the two groups [78, 79]. Disease relapse was not correlated with age, disease duration, MSCs source, CYC pre-treatment, baseline SLEDAI score, or proteinuria levels [59]. It was, however, found that patients who had higher levels of baseline IFN-γ and lower levels of baseline IL-6 showed a good clinical response to MSCT; serum TNF-α, IL-17, TGF-β1, and IL-10 had no difference between MSCT responders and non-responders among these patients [81]. This result is consistent with those of MSCT of RA [58].

Studies show that MSCT patients with lupus nephritis cannot get a positive therapeutic effect compared to placebo group [82] and are likely to cause renal insufficiency [59]. At present, there is still a big controversy about the therapeutic effect of MSC on LN. Traditional biomarkers for LN include dsDNA, complement, proteinuria, and active deposits. New biomarkers include MCP-1, NGAL, CXCL-10, CXCL-16, IL-6, IL-17, VCAM, TGF in urine -β1 mRNA and L-PGDS. Chemokines and inflammatory factors can better predict LN [83]. The result of a meta-analysis showed that the renal sclerosis score, ds-DNA, ANA, creatinine, urea nitrogen, proteinuria, IL-2, IL-12, IL-17 and IFN-γ cytokine levels of MSCT LN mice decreased, while IL-4, IL-6, IL-10, TGF-β, MCP-1, TNF-α cytokines and Th1, Th17, Treg cells are not significantly changed [84]. Tfh (CD4+CXCR5+PD-1+Tfh, CD4+CD44+CXCR5+PD-1+Tfh), B (GC B (B220+GL7 +), PC (B22010 CD138 +)) cells, which are associated with high concentrations of autoimmune antibodies and LN, are decreased in animal model after MSCT. While administration of hBMSC in the middle of the clinical phase of the disease is believed to suppress auto-Ab production and prevent the occurrence of LN, but does not reverse the progression of an ongoing nephritis, revealing a new mechanism for MSC to treat LN [27].

MSCT can make SLE patients survive up to 85% in 5 years, and the clinical markers of patients continue to improve during the follow-up period. There is no difference in the treatment of patients with bone marrow- or umbilical cord-derived MSCs. However, the pre-MSCT state of patients may affect the efficacy of MSCT. Like RA, patients with higher levels of IFN-γ or lower levels of baseline IL-6 at baseline showed a good clinical response to MSCT, and administration of hBMSC midway through the course of the disease can slow down nephritis and prevent development of LN.

## Inflammatory bowel disease (IBD)

Inflammatory bowel disease (IBD) is a term used to describe two chronic inflammatory conditions of the gastrointestinal tract, i.e., Crohn's disease (CD) and ulcerative colitis (UC). They are characterized by alternate episodes of inflammation remission and recurrences that often results into intestinal fistula and stenosis [85]. The factors that affect IBD are diverse and complex and are caused by genetic-environmental interactions. Central to this response is the homeostasis between intestinal immune cells and epithelial integrity, which is composed of epithelial and mesenchymal cells [86]. Once the mucosa regenerates, microbial translocation and further inflammation are prevented [87]. TNF receptor-1 signaling in epithelial cells and Toll-like receptor activation are crucial in IBD pathogenesis; NF-κB pathways, STAT3, YAP/Notch control the regeneration of epithelial cells [88, 89].

Currently, 2 coprimary end points are used by clinicians to determine the effectiveness of therapeutic interventions in patients with Crohn’s disease (CD): symptomatic remission and endoscopic remission. There is generally a lack of accepted biomarkers to facilitate regulatory decision-making [90]. At present, FC and CRP are in clinical trials. FC is being utilized as a diagnostic, prognostic, predictive, and pharmacodynamic/response biomarker, strongly correlated with endoscopy and post-operative disease recurrence [91]. The disadvantage of CRP as a biomarker is its poor specificity and so it’s often used in combination with other markers [90]. Other CD biomarkers being explored include[90]: inflammation markers such as IL-6 IL-22, IL-23; NGAL; miR-21, miR-31, miR-146a, and miR-375; TREM-1; pASCA; Oncostatin M; microbiome markers such as lower Firmicutes; higher Faecalibacterium; OmpC, ANCA, I2, A4-Fla2, Fla-X, Cbir1; and tissue injury markers such as Pro-C4, C4M, C3M, ECM1, BGM, EL-NE, C5M, Pro-C5, MMP-3, MMP-9, MMP-14. SPP24 whose level is related to endoscopy and is significantly different before and after treatment [92]. Matrix metalloproteinase-degraded type IV collagens, Serum oncostatin M, FC and CCR9 are considered to be biomarkers of drug efficacy [93, 94], while IFN reflects disease activity [95], and the transcription signals of CD4+T and CD8+T cells are closely related to the outcome [72, 95]. Both, phase I and II clinical studies done so far show that CRP and FC biomarkers are not affected by MSCT [96]. The predictive value of these markers for MSCT prognosis and outcome therefore still needs to be experimentally verified.

Given the expression of Toll-like receptors on the surface of MSCs, Kol et al. [97] found that bacteria can promote MSC to inhibit T cell proliferation. Studies have shown that in DSS model, tail vein injection of MSC increases the number of Ki-67( +) intestinal epithelial cells, LGR5( +) intestinal stem cells and CD31( +) endothelium in mouse intestinal, which demonstrated greater recovery of intestinal epithelial integrity compared with mesalamine group [38, 41]. Compared with control group, the infiltration of F4/80+ macrophages and monocytes in the colon tissue of MSC-DSS group was reduced, and FOXP3(+) Treg increased, but the number of CD3(+) T cells did not change significantly [41].

EF mice with administration of DF-MSCs exhibited outcomes, returned to normal body weight, healed the fistulas and significantly reduced mortality and prolonged survival [98]. Intravenous or intraperitoneal or locally injection of MSC significantly down-regulated IL-17A, IL-1β, IL-6, TNF-α, IFN-γ, CD8, IL-12, SAA and chemokines, while up-regulated IL-10 and TGF-β and the expression of VEGF and Ang-2 [13, 38, 98–100]. Inflammation-related ROS and lipid peroxidation product levels decreased, and the expression of antioxidant enzymes (Catalase and SOD1 not SOD2 increased) showed the reverse pattern as oxidative stress; MSC show the capacity of inhibiting Th1 and Th17 inflammatory cells, promoting Th2 and Treg cells [13, 38, 99, 100]. A large number of studies focus only on the changes of T cells, but few people pay attention to the activities of B cells related to antibody production. Chao et al. [101] noticed an increase in Treg and CD5+ Breg cells in MSCT group; IL-10 produced by Breg cells can correct the imbalance between Treg and Th17/Th1 cells.

Perianal fistula is a serious complication of CD. MSC is a new and promising treatment for perianal fistula due to its capacity for immune regulation and tissue repair. In one study, 12 weeks after MSC local injection, 40.0%, 80.0% and 20.0% of cohort 1 [1 × 10^7^ cells], 2 [3 × 10^7^ cells], and 3 [9 × 10^7^ cells] achieved full fistula closure [42]. And after 4 years, in cohort 2, all fistulas were closed. In this same 4 years, in cohort 1 had 63% of fistulas closed while and in cohort 3 had 43% of fistulas closed. None of the patients had detectable anti-HLA antibodies 24 weeks and 4 years after therapy [102]. Unlike in other diseases, MSCs are often transplanted directly into the patient's fistula tissue in IBD [42, 103–105], but blood transfusion can also be used [43, 96]. In addition to complete healing of the fistula for extended period of time following local MSCT, the levels of IL-8, IL-1β and IL-6 in the fistula tissue were significantly increased; however, there was no significant difference in these factors between normal tissue and fistula tissue after MSC treatment, accompanied by decreased PDAI, MSS, PDAI and increased SIBDQ, but no change in CDAI, CRP, fibrinogen, WBC [42, 104]. We have not found the number of Treg, the levels of CRP and CF have differences before and after intravenous infusion of MSC [96], and there was no significant difference in CRP between MSCT response group and non-response group [43].

Above studies show that MSC therapy can improve the quality of life of IBD patients. Intravenous administration of MSC restores immune function in mice, promote vascular regeneration, and reduce oxidative stress. These findings still need further studies to verify. Local injection of MSC promotes healing of fistula in patents with refractory Crohn's disease through immunosuppression and further promotes intestinal epithelial regeneration. Although CRP and CF are commonly used clinical biomarkers to reflect the disease activity of patients, evidence to support their use in MSC treatment monitoring is still lacking.

## Conclusions

These studies summarized above show that the active CID is caused by an abnormal immune homeostasis that can be restored by MSCT. MSCT reduces serum inflammation-related factors TNF-α, IFN-γ, IL-1β, IL-6, IL-8, ROS levels and the number of peripheral blood Th, Tfh, and B cells, while increasing IL-10, TGF-β, IDO (a key factor in MSC immunoregulation), angiogenic factors, and the number of Treg and Breg cells. Importantly, in RA, high serum level of IFN-γ in patients before mesenchymal stem cell transplantation and the transient increase in IFN-γ after transplantation indicate that the patient can achieve better results, due to its ability to promote the release of IDO to promote MSC’s immune activity; it was also found that patients with SLE who had higher levels of baseline IFN-γ and lower levels of baseline IL-6 showed a good clinical response to MSCT The combined use of cytokines including IFN-γ and MSC can enhance the response of patients treated with MSC, which indicates to a certain extent that patients with higher levels of cytokines in the serum treated with MSCs can achieve better results, or those who treated with these cytokines pretreated MSC may have a better effect.

## Prospect

At present, there are a large number of clinical studies looking at the changes that occur in patients before and after MSCT in order to find viable indicators of treatment outcomes. It is important that multiple immune cell subgroups, cytokines and clinical features are compared between patients that respond to MSC therapy and those that do not respond so as to have a clearer understanding of which biomarkers indicate response and which ones do not. This further enables objective exploration of mechanisms by which these factors affect MSC therapy.

The indicators to be considered for the inclusion of MSC treatment patients usually include: Disease Activity Score (DAS) 28, Simple Disease Activity Index (SDAI), and Clinical Disease Activity Index (CDAI) for Rheumatoid arthritis (RA); SLEDAI (Urine protein and creatinine clearance should be considered for LN) for Systemic lupus erythematosus (SLE) and CD Activity Index (CDAI) score of < 250 for Crohn’s disease (CD).

Treatment endpoint detection indicators often include: The remission rates of American College of Rheumatology (ACR) 20, ACR 50 and ACR 70, HAQ, RF, CRP, anti-citrulline antibody for Rheumatoid arthritis (RA); British Isles Lupus Assessment Group score (BILAG), SLEDAI, Quality of life Month SF-36, Quality of life EQ-5D, Steroids dose, and Lupus serology (Alb, ANA, dsDNA, C3, C4), Renal function (GFR, BUN, urinalysis) for Systemic lupus erythematosus (SLE), and monitoring of the closure of all treated external openings that were drained at baseline to determine fluid collections > 2 cm by masked central MRI, PDAI, CDAI, IBDQ, time to combined remission, relapse and time to relapse, and van Assche score for Crohn’s disease (CD). In the future, these markers can be used as clinical indicators and biomarkers for CID patients undergoing mesenchymal stem cell therapy (MSCT).

## Data Availability

Data sharing is not applicable to this article as no datasets were generated or analyzed during the current study.

## Abbreviations

CID: Chronic inflammatory disease
MSC: Mesenchymal stem cells
hUC-MSCs: Human umbilical cord mesenchymal stem (stromal) cell
adMSCs: Adipose-derived mesenchymal stem cells
BMSC: Bone marrow-derived mesenchymal stromal cells
MSCT: Mesenchymal stem cell transplantation
GvHD: Graft versus host disease
IDO: Indoleamine 2,3-dioxygenase
TGF-β: Transforming growth factor beta
PGE2: Prostaglandin E2
NO: Nitric oxide
RA: Rheumatoid arthritis
SLE: Systemic lupus erythematosus
IBD: Inflammatory bowel disease
PBMC: Peripheral blood mononuclear cell
SAA: Serum amyloid protein A
CRP: C reactive protein
Tfh: T follicular helper cells
Th: T helper cell
Breg: Regulatory B-cells
ROS: Reactive oxygen species
ALT: Alanine transaminase
ANC: Absolute neutrophil count
AST: Aspartate transaminase
BUN: Blood urea nitrogen
MTX: Methotrexate
WBC: White blood cell
WOMAC: Western Ontario and McMaster Universities Arthritis Index
VAS: Visual analogue scale
LDA: Low disease activity
LN: Lupus nephritis
P-MSCs: Placental-derived MSCs
EF: Enterocutaneous fistula
MSS: MRI score of severity
PDAI: Perianal disease activity index
CDAI: Crohn’s disease activity index
ADSVF: Adipose-derived stromal vascular fraction
SIBDQ: Small inflammatory bowel disease questionnaire
